# The value of broad taxonomic comparisons in evolutionary medicine: Disease is not a trait but a *state of a trait*!

**DOI:** 10.1002/mco2.174

**Published:** 2022-09-22

**Authors:** Mihaela Pavličev, Günter P. Wagner

**Affiliations:** ^1^ Department of Evolutionary Biology University of Vienna Vienna Austria; ^2^ Department of Ecology and Evolutionary Biology Yale University New Haven Connecticut USA; ^3^ Yale Systems Biology Institute Yale University West Haven Connecticut USA; ^4^ Department of Obstetrics Gynecology and Reproductive Sciences Yale School of Medicine New Haven Connecticut USA; ^5^ Department of Obstetrics and Gynecology Wayne State University Detroit Michigan USA

**Keywords:** comparative medicine, evolutionary medicine, pregnancy, variational traits

## Abstract

In this short paper, we argue that there is a fundamental connection between the medical sciences and evolutionary biology as both are sciences of biological variation. Medicine studies pathological variation among humans (and domestic animals in veterinary medicine) and evolutionary biology studies variation within and among species in general. A key principle of evolutionary biology is that genetic differences among species have arisen first from mutations originating within populations. This implies a mechanistic continuity between variation among individuals within a species and variation between species. This fact motivates research that seeks to leverage comparisons among species to unravel the genetic basis of human disease vulnerabilities. This view also implies that genetically caused diseases can be understood as extreme states of an underlying trait, that is, an axis of variation, rather than distinct traits, as often assumed in GWAS studies. We illustrate these points with a number of examples as diverse as anatomical birth defects, cranio‐facial variation, preeclampsia and vulnerability to metastatic cancer.

## Graphical abstract

Pathological variants are often extreme cases along lines of normal biological variation and can coincide with normal phenotypes of other species. For this reason, the comparative study of species can be informative of that of pathological variants.

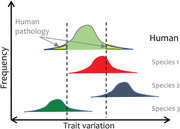

## 1 INTRODUCTION

Evolutionary biology has become an important conceptual and empirical contributor to biomedical research, leading to the growing field of evolutionary medicine.^1^, ^2^, ^3^, ^4^, ^5^ Given that the evolutionary past has shaped present‐day human development, physiology and behavior, evolution will also have influenced the vulnerability to and the nature of human disease. Some of the explanatory concepts that arose in this field are the *mismatch* between slowly evolving human biology and the fast‐changing modern environment,^6^ the *trade‐offs* (or compromise) among multiple traits under selection simultaneously, or the insight that the ultimate target of selection, the lifetime reproductive success, doesn't necessarily scale with overall health or longevity.^7^, ^8^ These concepts are parts of the now widely appreciated explanatory toolkit of evolutionary medicine. Thereby, the main body of work focuses on phenomena observable in the human branch of evolutionary history, whereas comparative approaches, although present (e.g., in cancer research^9^, ^10^, ^11^), are less frequent and less broadly appreciated. Restricting oneself to comparisons within the human lineage is often a plausible choice, because we have more information about the environment and lifestyle of past human populations than that of our earlier ancestors. What is more, some of the human traits are indeed lineage‐specific and thus not shared with other mammals and even less with other vertebrates. Examples are aspects of cognition, culture and social behavior. Such traits naturally cannot be studied outside the human lineage.

Yet a vast proportion of human traits, in particular most physiological, morphological and developmental traits, are widely shared with other mammals, amniotes, or even the whole vertebrate clade. To address the disease vulnerabilities of these traits, a broad taxonomic evolutionary approach to medicine can be an important resource. How broad comparative studies can contribute to finding the *mechanisms* of human disease and disease resistance is often not immediately apparent. Here, we briefly address this question both in theory as well as using empirical examples.

## 2 THE CONTINUITY OF NORMAL AND PATHOLOGICAL VARIATION WITHIN SPECIES: DISEASE IS NOT A SEPARATE TRAIT

Medicine and evolutionary biology share a fundamental interest, that is, the interest in phenotypic variation. Medicine is predominantly concerned with a particular portion of human phenotypic variation, namely deleterious variation, that is, disease (for discussions on defining disease see^12^, ^13^, ^14^). Similar to medicine, evolutionary biology is a scientific field dedicated to the study of variation, but irrespective of its health effects. In this case, the full range of biological variation, its patterns within and among species, its change through evolutionary time, its underlying causes and effects are under investigation. The two fields thus share a core research interest. Not surprisingly, therefore, many concepts and tools developed in evolutionary biology can be useful in biomedical research. One such concept is the distinction between a trait, say body height, and trait state, say body height of 170cm. Biological variation often (but not always) can be described as different states of a unitary biological trait.

**FIGURE 1:**
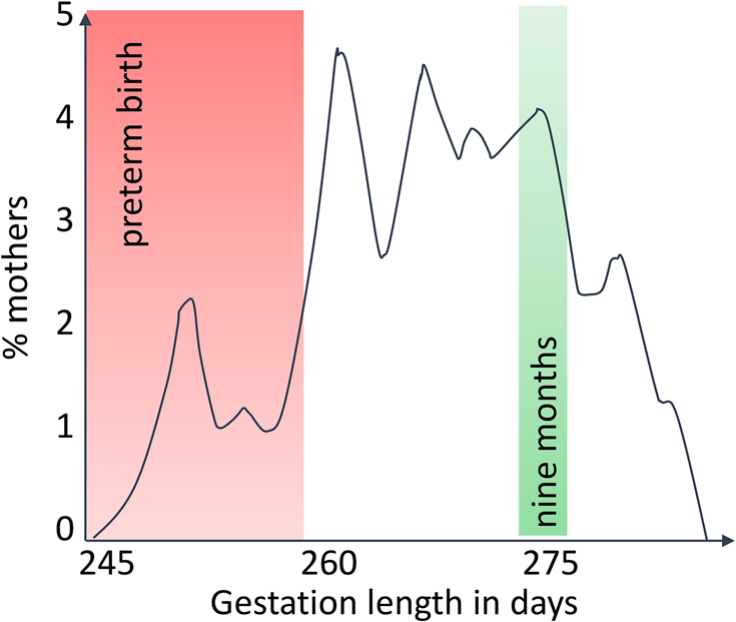
Distribution of gestational lengths in humans. Modified from Jukic et al 2013.^15^ (Data: N Carolina Early Pregnancy Study, *N* = 125, ovulation‐based measurement.)

Figure 1 illustrates this point using the distribution of gestational lengths in human population.^15^ The distribution is broad, with a large percentage of women birthing before or after the defined normal, “term,” window. Not all this variation is genetically heritable; however gestational length clearly has a genetic component. Guided by the consequences for the survival and life quality of the neonate, medicine has defined birth occurring before 37 weeks of gestation as “preterm birth.” The pathological portion of variation is a part of the distribution of an underlying biological trait “*gestational length*.” Prematurity does not have a separate genetic basis from gestational length, rather its genetic basis is largely due to alleles at the same genes as that of other realizations, or *states*, of gestational length.^16^ In other words, any alleles that shorten gestation can also move an individual over that pathological limit, depending on where in the distribution the individual is located due to her other alleles. This explains why the same genetic variants can be sometime deleterious and sometime not.

**FIGURE 2:**
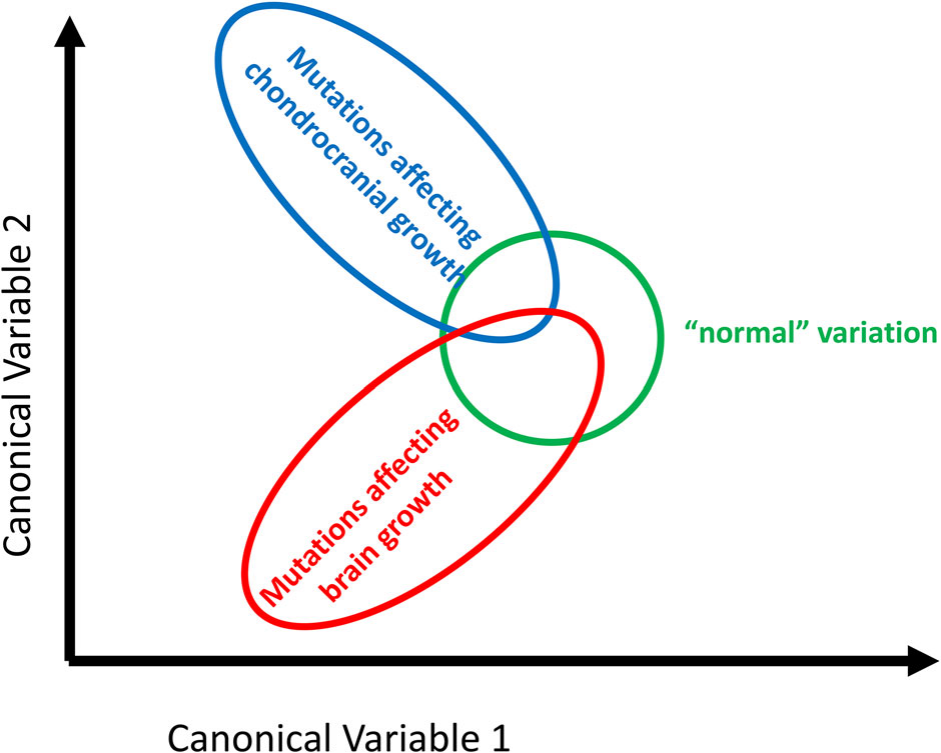
Schematic representation of the results of a study of mutations affecting craniofacial shape in mice^17^. The majority of mutations cluster tightly among normal variation, but two classes of mutations reveal two distinct dimensions of craniofacial variation, those affecting cartilage and replacement bones and those affecting brain growth.

To illustrate the importance of distinguishing traits as directions of phenotypic variation from trait states as one particular realization of a trait, we refer to a recent study of genes affecting cranio‐facial development in mice.^17^ In an analysis of cranio‐facial shape variation the authors found that the majority of genetic effects are affecting a limited number of phenotypic directions, out of the many possible dimensions of skull shape. This reflects a limited number of developmental pathways through which the genetic polymorphisms exert their effects on the phenotype. From this the authors conclude that certain outcomes, including those classified as developmental defects, result from effects on a fixed coordinated set of developmental processes. For instance, cranio‐facial shape is to a large degree influenced by the growth of the skull base (chondrocranium) in relation to the growth of the brain (Figure 2). This is true for humans and mice^17^ and likely also true for most mammals. Skull base growth is determined by the growth of the chondrocranium (trabeculae) and thus affected by loci influencing cartilage development and growth. In contrast, brain growth is affected by genes involved in neurogenesis and neuron differentiation. It is thus more meaningful to consider these directions of variation as the *traits* (chondrocranium vs. brain) and any outcome along these lines as *states of these traits*, including so‐called birth defects such as achondroplasia characterized by deep set eyes and short facial features relative to the cranial vault.

Many human diseases therefore can be defined as extreme portions of the possible range of variation that human anatomical and physiological traits can take. To understand their etiology means to understand the causes of variation in a physiological or developmental trait, including those across a trait's healthy range. One consequence of this perspective is that for many diseases we do not find a genetic basis that is separate from that of the normal trait. It is thus misleading to call a particular disease a “complex trait” since it implies that the disease is a different biological entity than the normal traits of an organism. In the next section, we will argue that this way of thinking also extends to differences between species, because species share, through their common evolutionary history, many traits (e.g., placentation in mammals) even though they differ in the specific expression of these shared traits (e.g., the depth of placentation).

Following the argument above one also should reflect on the question whether all diseases can be conceptualized as extensions of normal biological variation. We feel that this question is not clearly resolved, but candidates of conditions that may not fit this model are radical deviations of healthy organization of the body, such a cancer and degenerative diseases, often detected as mutations of major effects.

## 3 COMMON AXES OF VARIATION WITHIN AND AMONG SPECIES

Having argued for the continuity between normal and disease variation within species, one further evolutionary principle important in this context is the continuity of variation within and among species, that is, the fact that differences among species arise as differences among individuals within a species.^18^ Branches of evolutionary biology that are concerned with within‐species or among‐species differences ask somewhat different questions and use different tools, contributing to the perception of a conceptual discontinuity between fields. Variation within species is studied by population and quantitative genetics. The tools of these fields are already well integrated into medical research (e.g., GWAS, epidemiology). Variation among species is studied by the comparative method,^19^, ^20^ an approach substantially less well integrated into medical research. Understandably so, as at first it appears rather unlikely that a broad comparative analysis of, for example, pregnancy from opossum to mouse and human may reveal anything useful about pregnancy complications in women. Below we explain why this view is mistaken.

The shared underlying pathways of trait development explain why genetic differences among species for a particular trait can point to the same genes and molecular pathways as variation between individuals within species (see below for studies showing similar genetic basis of preeclampsia and species differences in placental phenotype). This observation further underscores the value of understanding normal variation among species for understanding the mechanistic basis of disease.^21^, ^22^

Differences among species arise from mutations that first appear as differences among individuals within a species. As species diverge, these differences may become fixed in one or the other newly arising species. As long as the underlying developmental mechanisms producing the trait remain the same, that is, the traits are homologous among species, the kind of variation caused by mutations often occurs along similar phenotypic lines in different species. That is, a fundamental continuity between variation within and among species can be expected (especially within taxonomic groups with common body plan, such as in mammals) and studying one can therefore inform the other. We take advantage of this continuum when we use comparisons across a range of species to understand how the pathological variation may arise within species.

**FIGURE 3:**
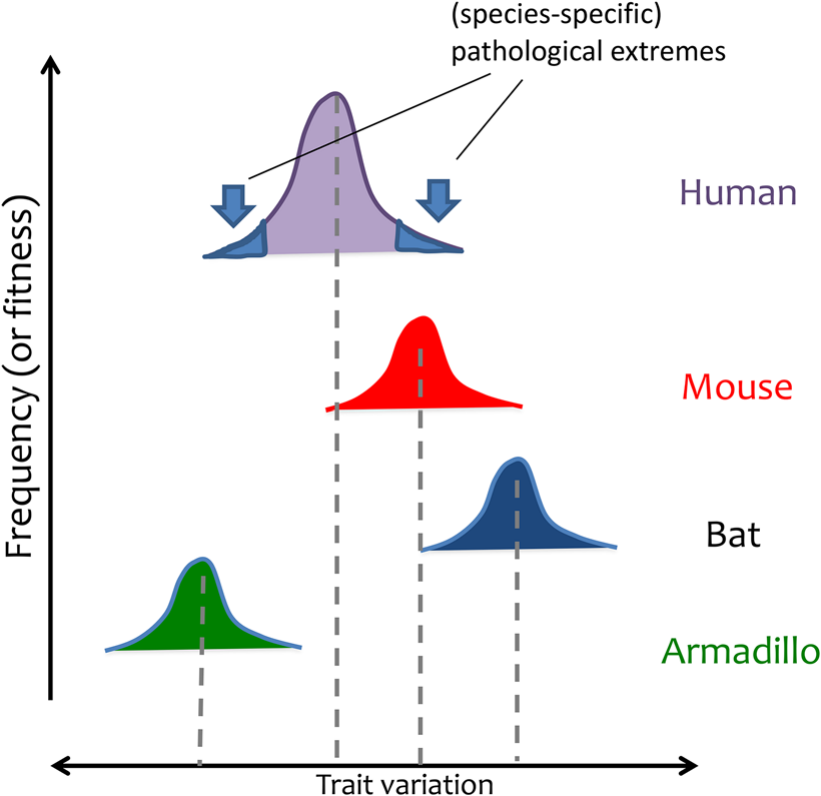
Common axis of trait variation across species, with species‐specific optima. The main axis of variation is often shared across species, with species‐specific distributions. Thus, the optimum in one species can be a pathological extreme in another.

Indeed, in traits shared with other species, it is repeatedly found that the genetic basis underlying differences among species is also responsible for variation within species (examples below). This is implicitly acknowledged by the widespread use of animal models in biomedical research, in which the researcher relies on the assumption that phenotypically similar traits among species are due to conserved underlying pathways. Note that different species may show optima at different values of a trait, even if sharing the same axis of variation, and therefore the part of variation that is considered pathological in one species is not necessarily pathological in other species (Figure 3).

**FIGURE 4:**
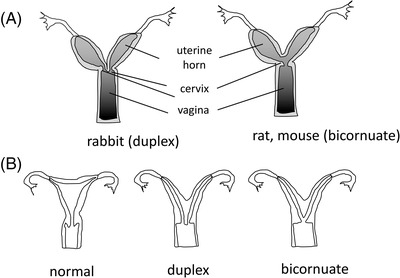
The parallel between among‐species’ normal states and within‐species pathological states. (A) uterine anatomy across different species varies in the degree of fusion of Mullerian tube derivatives (uterine horns). (B) Human (within‐species) variation includes pathological anatomy of a similar kind as those normal in other species.

Some examples will illustrate the point. One excellent illustration comes from the well‐known anatomy of the female reproductive tract. This anatomical region is widely variable across mammals (Figure 4A). Interestingly, human congenital uterine variations recapitulate a similar spectrum of variation (Figure 4B).^23^ In this case, the explanation is well understood; it is due to the fact that the female reproductive tract arises in development from a pair of embryonic structures, called the Mullerian ducts. The uterus of humans and apes, called the uterus simplex, arises through the fusion of the distal parts of the Mullerian tubes. During human development, perturbations of this fusion process creates a spectrum of morphologies ranging from uterus duplex (i.e., two uterine tubes complete with two cervices, like in the opossum), the bi‐cornute uterus (consisting of two “uterine horns” united posteriorly in a small uterine corpus like in mouse and many other mammals) to the bi‐partite uterus where there is a septum separating left and right parts of a unitary uterus. This parallel between pathological variation within our species, and divergence between species, arises because the process of female reproductive tract development is shared across species, and only the degree of fusion evolved to be species‐specific.

**FIGURE 5:**
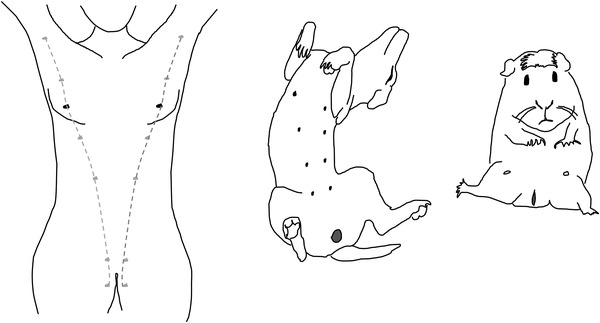
The milk ridge and the location of mammaries. The milk ridge, as a developmental trait, is present in all eutherian mammals, but the number and location of realized mammaries is different in different species. However, in humans, supernumerary nipples can occur along the milk ridge line due to incomplete developmental regression of the ridge.

Another, similar, example is the variation in the placement of supernumerary nipples in humans (not just women). Such supernumerary nipples appear along an imaginary line extending from the axillar to the inguinal region (Figure 5).^23^ Supernumerary nipples are not usually deleterious in humans, yet they are a case of deviation from normal development. The placement of normal nipples and teats among eutherian mammals (aka placental mammals) follows the same pattern; with primates having thoracic mammae, the dugong having axillar mammae and the cow inguinal. The reason for this parallelism is again a shared developmental pathway. In eutherian mammals, including humans, the embryo develops a paired crest of epidermal tissue capable to differentiate into mammary glands, the so‐called “milk ridge.” The species‐specific location of mammaries arises through differential degeneration of parts of the milk ridge leaving only those parts in place that develop into the species‐specific pattern of mammary placement. In humans, incomplete regression of the milk ridge thus leads to developmental variants that are broadly parallel to those among different species of eutherian mammals. Our insistence on speaking of eutherian mammals here is because marsupials do not have a milk ridge, but a broad pad of competent tissue developing into mammary glands at either side, as well as in the midline, while eutherian mammals never have mammaries placed on the midline.

An example that directly addresses the genetic basis of variation among and within species is the work of Mick Eliot and Bernard Crespi on the genetic basis of placental phenotype among mammals and preeclampsia in humans.^24^ Preeclampsia is a consequence of the insufficient invasion of the uterine endometrium by the placenta. Invasiveness of placentation also varies widely across placental mammals, from very superficial placentation in hoofed animals, over intermediate (endotheliochorial) placentation in carnivores, to highly invasive placentation like in human and mice. Phylogenetic analyses show that placentation was invasive in the common ancestor of all eutherian mammals.^25^, ^26^, ^27^ As a consequence, all extant eutherian species with superficial placentation must have secondarily evolved this characteristic. Elliot and Crespi^24^ have used this information to study what evolutionary changes occurred in three lineages that evolved reduced invasiveness, the kangaroo rat, the tree shrew, and the clade uniting lemurs and galagos. By determining which genes were under selection in these lineages, they recovered many genes with alleles associated with preeclampsia in humans. In their study they identified many more genes, which are candidates for potentially contributing to preeclampsia. Their result means that the genetic basis of placental invasiveness across species is highly similar to that within human species. An interesting extension of this result is to ask why lemurs, galagos, and cows do not get preeclampsia, even though their placentation is even less invasive than that of preeclampsia patients. Researching this question in these species holds the promise to learn how to prevent the deleterious consequences of shallow placentation for the mother.

Yet another example connects the genetics of mammalian placental invasiveness and the vulnerability to cancer metastasis. The invasiveness of the placenta and a species’ vulnerability to cancer^28^, ^29^ are positively correlated, such that species that evolved lower placental invasiveness are also less likely to develop malignant cancer. Anecdotal evidence for this fact is provided by case studies from the veterinary literature. For instance a case report out of India documents a cow with a large melanocytoma (∼1lb) without any signs of metastatic disease.^30^ This fact can in part be explained by differences in the invasibility of stromal tissue, both in skin as well as in the uterus. In vitro invasibility experiments show that human skin and endometrial fibroblasts are more readily invaded by cancer and trophoblast cells than bovine fibroblasts.^31^ Identifying the gene expression differences between human and bovine fibroblasts affecting stromal invasibility identifies genes also known to affect patient outcome. For instance, the expression of TGFb1 in stromal fibroblasts is correlated with higher invasibility by both, cancer cells as well as trophoblast cells, and is also associated with worse outcome for cancer patients.^32^ That this pattern is not only anecdotal has been show in recent studies comparing genes associated with placental phenotype and cancer malignancy across a sample of mammalian species.^33^, ^34^ That these insights do hold translational potential is shown by the fact that modulating gene expression in human cells to make them more like bovine fibroblasts can increase their ability to resist cancer cell invasion.^31^

## 4 VARIATIONAL VERSUS MECHANISTIC ARCHITECTURE OF TRAITS

A frequent source of misperception is the difference between genetic architecture of a trait, as revealed by population genetic methods, and the mechanistic pathways underlying a trait. A shared mechanistic basis of a trait does not mean that exactly the same loci will be detectable by association with variation in this trait. Only those loci polymorphic in a population can be detected by GWAS or other genetic association methods. For instance, body size is a complex trait that is due to the activity of many molecular and physiological pathways in which the products of a large number of genes participate. Body size variation thus can potentially be influenced by variation in any of these genes, besides contributions from the environment. In a GWAS, heritable size variation is detected that is due to variation in some subset of genes that happen to be polymorphic in the investigated population. Such subsets of polymorphic loci can differ between populations and species—even when the trait shares the same mechanistic pathways. Consequently, the “*genetic architecture” of trait variation* can be different between populations and species even if the physiological pathways leading to the trait are the same. Thus, it is not surprising that GWAS studies of body size variation, for example in mice and rats, will detect different loci, because there is no reason to expect that the same subset of genes responsible for body growth should be polymorphic in these two species. Polymorphic loci detected in genetic mapping studies can point to the mechanistic pathways regulating and realizing a trait, for example, body size or gestational length, but in any one population only a sub‐set of these genes will be polymorphic and thus detectable by genetic mapping. Hence, the genetic basis of trait variation is not the same as the genomic basis of the trait itself.

## 5 CONCLUSION

Both medicine and evolutionary biology are sciences of biological variation and thus have a natural affinity in their concepts and approaches. In this contribution, we build on evolutionary approaches to emphasize two aspects of variation that we consider important in searching for the genetic bases of diseases. The first is that the pathological and healthy variation often are parts of a continuum, that is, the disease is an extreme state of a trait, rather than itself a “distinct complex trait.” The consequence is that understanding of genetic basis of healthy, nondeleterious, variation may contribute to the understanding of genetic basis of its deleterious extreme, the disease. Second, we explain and illustrate that the continuity of general developmental genetic pathways reaches across species boundaries. Comparative study of trait variation across species can thus contribute to understanding human variation, including pathological variation. Beyond understanding the genetic basis of diseases, the fact that different species established the states of the shared trait, which correspond to deleterious states in humans, can offer rich insights into the mechanisms of how to overcome human disease states.

## AUTHOR CONTRIBUTIONS

Mihaela Pavličev and Günter P. Wagner developed the ideas and wrote the paper.

## CONFLICT OF INTEREST

Coauthor GPW is an editorial board member of MedComm. GPW was not involved in the journal's review of, or decisions related to, this manuscript. MP declares no conflict of interest.

## ETHICS STATEMENT

No research activity for this manuscript requires an ethics approval.

## Data Availability

No original data are associated with the paper.
